# MPV17 Prevents Myocardial Ferroptosis and Ischemic Cardiac Injury through Maintaining SLC25A10-Mediated Mitochondrial Glutathione Import

**DOI:** 10.3390/ijms251910832

**Published:** 2024-10-09

**Authors:** Tao Xu, Guilan Chen

**Affiliations:** Instrumental Analysis Center, Qingdao Agricultural University, Qingdao 266109, China; xutao20180216@126.com

**Keywords:** ischemia/reperfusion injury, iron overload, myocardial ferroptosis, MPV17, mitochondrial glutathione

## Abstract

Ferroptosis is a recently identified iron-dependent programmed cell death with lipid peroxide accumulation and condensation and compaction of mitochondria. A recent study indicated that ferroptosis plays a pivotal role in ischemic cardiac injury with the mechanisms remain largely unknown. This study demonstrates that when an iron overload occurs in the ischemia/reperfusion cardiac tissues, which initiates myocardial ferroptosis, the expression levels of mitochondrial inner membrane protein MPV17 are reduced. Overexpression of MPV17 delivered via adenovirus significantly reduced ferroptosis in both cardiomyocytes with high levels of iron and cardiac I/R tissues. Mitochondrial glutathione (mtGSH), crucial for reactive oxygen species scavenging and mitochondrial homeostasis maintenance, is depleted in myocardial ferroptosis caused by iron overload. This mechanistic study shows that MPV17 can increase mitochondrial glutathione levels through maintaining the protein homeostasis of SLC25A10, which is a mitochondrial inner-membrane glutathione transporter. The absence of MPV17 in iron overload resulted in the ubiquitination-dependent degradation of SLC25A10, leading to impaired mitochondrial glutathione import. Moreover, we found that *MPV17* was the targeted gene of Nrf2, which plays a pivotal role in preventing lipid peroxide accumulation and ferroptosis. The decreased expression levels of Nrf2 led to the inactivation of MPV17 in iron overload-induced myocardial ferroptosis. In summary, this study demonstrates the critical role of MPV17 in protecting cardiomyocytes from ferroptosis and elucidates the Nrf2-MPV17-SLC25A10/mitochondrial glutathione signaling pathway in the regulation of myocardial ferroptosis.

## 1. Introduction

Cardiac diseases, including the ischemic cardiac injury, are principally caused by myocardial cell death [1,2,3,4]. Ferroptosis is a special form of programmed cell death, initially induced by several small compounds such as erastin or RSL3. This type of cell death is also marked by lipid peroxide accumulation and mitochondrial shrinkage [5]. Increasing evidence indicates that ferroptosis promotes cardiac injury in cardiac diseases, and therefore, targeting ferroptosis represents a promising approach for disease treatment [6,7]. Nonetheless, the mechanisms regulating myocardial ferroptosis remain poorly understood.

Iron overload is considered a critical factor in cardiac pathological processes, including ischemic cardiac injury [8,9,10,11,12,13]. In both clinical practices and the experimental mouse model, ischemic cardiac injury is usually accompanied by iron overload, which is an active participator in ferroptosis initiation [14,15]. Generally, iron overload is associated with the lipid peroxide build-up through the Fenton reaction at the onset of ferroptosis [16,17]. Moreover, alterations in the antioxidant status have also been implied in the initiation of ferroptosis during iron overload-related pathological processes [18,19]. In cardiopathy induced by doxorubicin (DOX), increased expression of Hmox1 degrades heme, resulting in the release of iron, consequent iron overload, and subsequent myocardial ferroptosis [20]. Here, we will further explore the signaling pathways regulating iron overload-induced ferroptosis in ischemic cardiac injury.

MPV17, a nuclear-encoded transmembrane protein located within the inner mitochondrial membrane, is known to be involved in mitochondrial DNA replication. Mutation in MPV17 usually leads to mitochondrial DNA deletion-associated diseases, such as neurohepatopathy [21,22]. The function of MPV17 is also closely related with mitochondrial oxidative phosphorylation, mitochondrial permeability transition pore (MPTP) regulation, and mitochondrial reactive oxygen species (ROS) metabolism [23]. Additionally, nuclear magnetic resonance (NMR) structural analysis suggested that MPV17 might act as a scaffold protein, whose function is not fully understood [24]. The deletion of MPV17 did not significantly impact cardiac functions under normal conditions; however, MPV17 could improve cardiac functional recovery following ischemia/reperfusion (I/R) injury [24]. This work aims to delve into the function of MPV17 in myocardial ferroptosis and its underlying molecular mechanisms.

Mitochondria serve not only as energy providers but also as signaling centers in various biological processes. Evidence suggests that the dysfunction of mitochondria promotes ferroptosis initiation [25]. The mitochondrial ROS generated by the activity of the α-ketoglutarate dehydrogenase complex is a causative factor in ferroptosis initiation in situations where cysteine is deprived. The depletion of mitochondria through Parkin-mediated mitophagy in human fibrosarcoma cells showed high resistance to cysteine deprivation-induced ferroptosis [25]. Another study shows that mitochondrial glutathione (mtGSH) functions as an antioxidant to eliminate oxidized lipids and maintains redox homeostasis in mitochondria [26]. The evidence indicates that a reduction in mtGSH makes cardiomyocytes more sensitive to ferroptotic stimuli [27]. However, mitochondria cannot synthesize GSH, but can import GSH from cytosol where all cellular GSH is synthesized [28]. Solute carrier family 25 member 10 (SLC25A10) is one of the key mtGSH transporters embedded in the inner mitochondrial membrane, responsible for importing GSH from the cytosol into the mitochondrial matrix [29]. The protective role of SLC25A10 has been revealed in RSL3-induced myocardial ferroptosis through importing GSH from cytosol into mitochondria [27]. We supposed that SLC25A10 also functions in iron overload-induced myocardial ferroptosis.

Nuclear factor erythroid 2-related factor 2 (Nrf2) is a transcriptional factor, which plays an important role in the clearance of lipid peroxides through controlling the expression of antioxidant genes. Numerous studies indicate that Nrf2 plays a potent role in the inhibition of ferroptosis, and targeting Nrf2 is a promising therapeutic approach for treating ferroptosis-driven diseases [30,31]. However, its targeted genes are not fully uncovered. This study has explored the regulatory effect of Nrf2 on MPV17 expression in iron overload-induced ferroptosis, aiming to identify potential targets of Nrf2 to further explore the mechanism of the inhibition of ferroptosis.

## 2. Results

### 2.1. MPV17 Prevented Iron Overload-Induced Myocardial Ferroptosis

**Figure 1:**
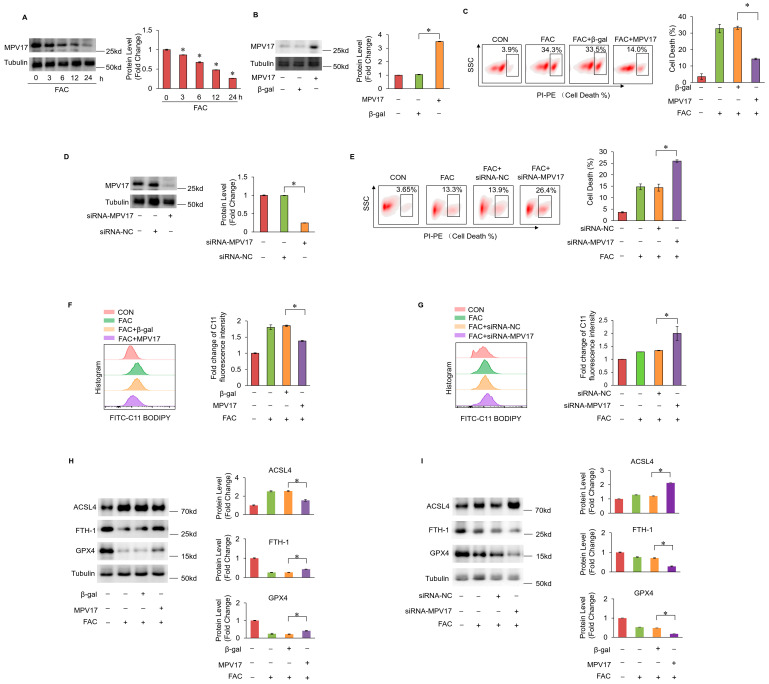
(**A**) Protein levels of MPV17 in cardiomyocytes treated with FAC evaluated by WB; (**B**) the overexpression of MPV17 in cardiomyocytes was detected by WB; (**C**) cell death labeled with PI staining in each group; (**D**) the knockdown of MPV17 in cardiomyocytes by siRNA was detected by WB; (**E**) cell death labeled with PI staining in each group; (**F**) C11-BO was applied to label the lipid peroxides and flow cytometry was used for the detection of green fluoresce in each group; (**G**) C11-BO was applied to label the lipid peroxides and flow cytometry was used for the detection of green fluoresce in each group; (**H**) ACSL4, FTH–1 and GPX–4 were evaluated by WB; (**I**) ACSL4, FTH–1 and GPX–4 were evaluated by WB; *, *p* < 0.05 was statistically significant.

To investigate the molecular mechanism of myocardial ferroptosis induced by iron overload, ferric ammonium citrate (FAC) was introduced into the culture medium to mimic the high-iron condition, thus inducing myocardial ferroptosis [32]. Under the treatment of FAC, the expression levels of MPV17 decreased in cardiomyocytes (Figure 1A). To investigate the function of MPV17 in regulating ferroptosis, we overexpressed MPV17 using an adenovirus carrying the *MPV17* gene in cardiomyocytes (Figure 1B). We found that the overexpression of MPV17 significantly prevented iron overload-induced ferroptosis (Figure 1C). Then, siRNA was applied to knockdown the endogenous MPV17 in cardiomyocytes (Figure 1D). The knockdown of MPV17 enhanced ferroptosis induced by FAC in cardiomyocytes (Figure 1E). To further explore the role of MPV17 in myocardial ferroptosis regulation, we detected the impact of MPV17 on lipid peroxide accumulation [33]. Our results demonstrated that treatment with FAC led to lipid peroxide accumulation, which was mitigated by MPV17 overexpression (Figure 1F). The knockdown of MPV17 exacerbated the accumulation of lipid peroxidation (Figure 1G). Next, we detected the impact of MPV17 on the expression of ferroptosis-associated proteins, including ACSL4, FTH–1 and GPX4 in cardiomyocytes treated with FAC [34]. The increased protein levels of ACSL4 and the decreased protein levels of FTH–1 and GPX4 indicated the initiation of ferroptosis, which were partially restored by MPV17 overexpression (Figure 1H). Moreover, the knockdown of MPV17 further promoted the expression of ACSL4 and decreased the expression of GPX4 and FTH–1 in cardiomyocytes treated with FAC (Figure 1I). All these results demonstrated that MPV17 prevented iron overload-induced ferroptosis.

### 2.2. MPV17 Prevented Myocardial Ferroptosis during Cardiac Ischemia/Reperfusion (I/R) Injury

**Figure 2:**
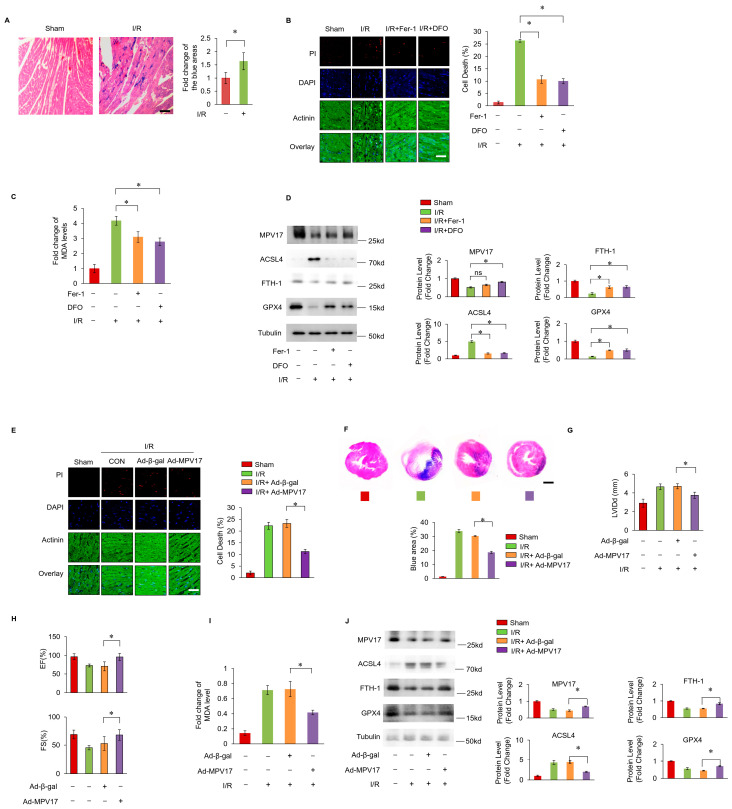
(**A**) Prussion Blue staining for the examination of iron levels in cardiac tissues; scale bar, 60 µm; (**B**) cell death labeled with PI staining in cardiac tissues after I/R surgery; red, PI; blue, DAPI; green, Actinin; scale bar, 60 µm; (**C**) statistic data of the relative MDA levels; (**D**) the expression of ACSL4, FTH–1 and GPX–4 were checked by WB; (**E**) cell death labeled with PI staining in cardiac tissues after I/R surgery; red, PI; blue, DAPI; green, Actinin; scale bar, 60 µm; (**F**) Masson trichrome staining for the examination of collagen in the heart tissue; (**G**) LVIDd, diastolic left ventricular internal diameter; (**H**) EF, the ejection fraction of the left ventricular diameter; FS, the fractional shortening of the left ventricular diameter; (**I**) statistic data of the relative MDA levels in each group; (**J**) the expression of MPV17, ACSL4, FTH–1 and GPX–4 were checked by WB; *, *p* < 0.05 was statistically significant; ns, none significance.

The iron levels detected by Prussian Blue staining increased in the I/R cardiac tissues, indicating that iron overload occurred when mice hearts underwent cardiac I/R injury (Figure 2A). Myocardial cell death dramatically increased in the mice hearts that underwent I/R injury, while the ferroptosis inhibitor ferrostatin–1 (Fer–1) or iron chelator deferoxamine (DFO) treatment decreased this cell death (Figure 2B,C). Then, lipid peroxide accumulation was examined by detecting the malondialdehyde (MDA) levels. The MDA levels were increased during the I/R injury, which were reversed by Fer–1 or DFO treatment. Moreover, the expression of ACSL4 was increased while the expression of FTH–1 and GPX4 were decreased, which were also reversed by Fer–1 or DFO treatment. All this evidence illustrates that iron overload-induced ferroptosis occurs during cardiac I/R injury. Next, we examined the role of MPV17 in myocardial ferroptosis in mice hearts that underwent I/R injury. The PI staining results of the cardiac tissues showed that the MPV17 overexpression mice group significantly reduced the myocardial ferroptosis during cardiac I/R injury compared with the β-gal overexpression group (Figure 2E). Moreover, the increased MDA levels in ischemic cardiac tissues were attenuated by MPV17 overexpression (Figure 2I). The increased expression levels of ACSL4 and the decreased expression levels of FTH–1 and GPX4 were also reversed in the MPV17 overexpression group (Figure 2J). Myocardial fibrosis is closely related to cardiac remodeling and the recovery of long-term function after I/R injury. We found that the collagen areas were decreased by the MPV17 overexpression after cardiac I/R injury. Moreover, the diastolic left ventricular internal diameters (LVIDd), the ejection fraction of the left ventricular diameter (EF) and the fractional shortening of the left ventricular diameter (FS) were also partially recovered by the MPV17 overexpression, indicating that MPV17 improved cardiac remodeling after I/R injury (Figure 2G,H). All this evidence demonstrated that MPV17 could prevent myocardial ferroptosis during cardiac I/R injury.

### 2.3. MPV17 Prevented Iron Overload-Induced Ferroptosis through Maintaining Mitochondrial Glutathione (mtGSH) Levels

**Figure 3:**
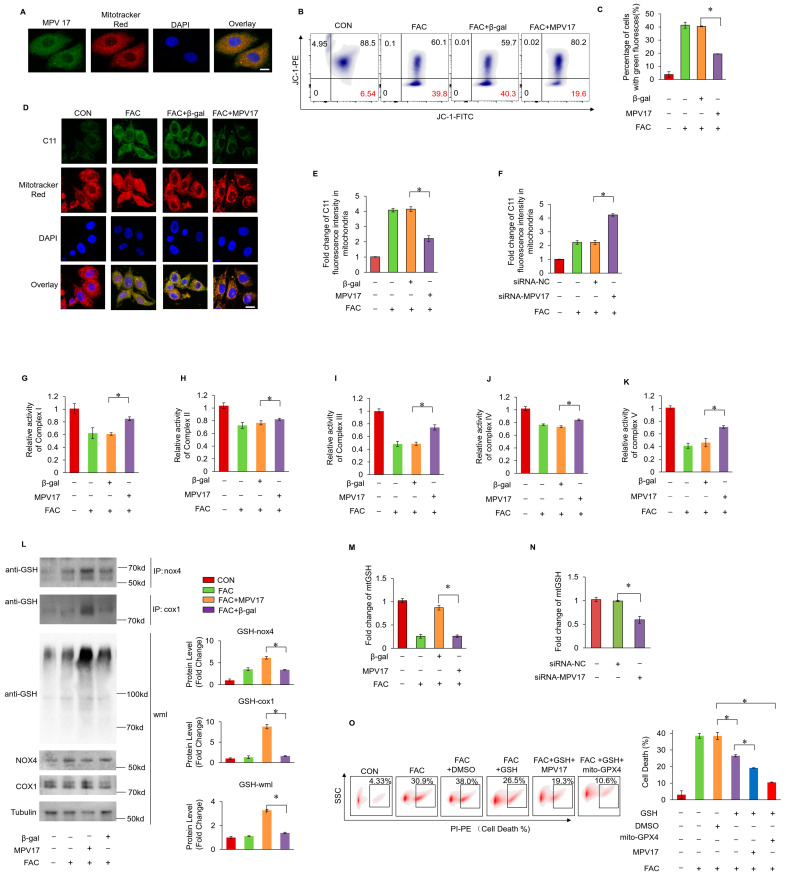
(**A**) The localization of MPV17 detected by immunofluorescence and confocal imaging; green, MPV17; red, mitotracker; blue, DAPI; scale bar, 10 µm; (**B**) JC–1 and flow cytometry were used for the measurement of mitochondrial membrane potential; (**C**) the ratio of cells with green fluorescence in each group labeled with JC–1; (**D**) confocal imaging of the lipid peroxides labeled with C11-BO; green, C11-BO; red, mitotracker; blue, DAPI; scale bar, 20 µm; (**E**) fold changes in the green fluorescence intensity of the C11-BO staining in mitochondria in each group; (**F**) fold changes in the green fluorescence intensity of the C11-BO in mitochondria in each group. (**G**–**K**) The relative activity of the Complex I-V; (**L**) the glutathionylation of nox4, cox1 and the whole mitochondria lysates (wml) were evaluated by WB. (**M**) the percentage of cell death labeled with PI staining in each group. (**N**) Fold change in the mtGSH levels in the cardiomyocytes in each group. (**O**) Fold change in the mtGSH levels in the cardiomyocytes in each group; *, *p* < 0.05 was statistically significant.

It was hypothesized that mitochondrial dysfunction was critical in the initiation of ferroptosis. The immunofluorescence staining analysis indicated that MPV17 was localized within the mitochondria, indicating its regulatory role in mitochondrial homeostasis (Figure 3A). Firstly, we detected the mitochondrial membrane potential though the JC–1 staining and flow cytometry. The decreased mitochondrial membrane potential was observed under FAC treatment, while the concurrent overexpression of MPV17 partially restored mitochondrial membrane potential (Figure 3B,C). Then, we detected the distribution of lipid peroxides through the C11-BO staining and confocal imaging. It was observed that lipid peroxides mainly accumulated in the mitochondria of cardiomyocytes treated with FAC, which indicated the critical role of mitochondria in ferroptosis initiation (Figure 3D). The overexpression of MPV17 prevented lipid peroxide accumulation in the mitochondria of cardiomyocytes treated with FAC, while the knockdown of MPV17 promoted lipid peroxide accumulation in the mitochondria of cardiomyocytes treated with FAC (Figure 3D–F). These results illustrated that MPV17 prevented mitochondrial lipid peroxide accumulation in cardiomyocytes administrated with FAC. Next, we detected the impact of iron overload on the mitochondrial respiratory chain. FAC treatment significantly disturbed the activity of the mitochondrial respiratory chain (Complex I–V) while the overexpression of MPV17 partially recovered the activity of mitochondrial respiratory chain (Figure 3G–K). Although the mitochondrial Fe^2+^ levels were significantly increased, detected by the mito-FerroGreen staining after the treatment of FAC, the overexpression of MPV17 did not influence the mitochondrial Fe^2+^ levels (Supplementary Figure S1). Then, we detected its impact on the maintenance of mitochondrial redox homeostasis. mtGSH has been confirmed to play an essential role in preventing ferroptosis initiation [27]. We found that FAC treatment decreased the levels of mtGSH, while the overexpression of MPV17 partially recovered mtGSH levels (Figure 3M). The knockdown of MPV17 promoted the exhaustion of mtGSH in cardiomyocytes treated with FAC (Figure 3N). mtGSH was not only an antioxidant reagent but was also essential for mitochondrial protein glut.athionylation, which protected proteins from oxidative damage under oxidative stress. We found that mitochondrial protein glutathionylation increased slightly in cardiomyocytes treated with FAC, while the overexpression of MPV17 further promoted mitochondrial protein glutathionylation. Interestingly, the core enzymes of the mitochondrial respiratory chain, NADPH oxidase 4 (nox4) and cytochrome c oxidase 1 (cox1), were also glutathionylated, which were further promoted by MPV17 overexpression. The glutathionylation of nox4 and cox1 protected them from oxidative damage and explained the recovery of mitochondrial respiratory chain activity after MPV17 overexpression. To further confirm the role of mtGSH in iron overload-induced ferroptosis, we pre-treated the cardiomyocytes with GSH for 6 h. We found that GSH treatment prevented iron overload-induced ferroptosis, and the simultaneous overexpression of a mitochondrial GPX4 (mtGPX4) further reduced myocardial ferroptosis (Figure 3O). However, the simultaneous overexpression of MPV17 was not as pronounced as the simultaneous overexpression of mtGPX4 in the reduction of ferroptosis in cardiomyocytes treated with GSH (Figure 3O). These results indicated that MPV17 functioned upstream of the GSH/GPX4 signaling. In conclusion, iron overload decreased mtGSH, which led to mitochondrial lipid peroxide accumulation and mitochondrial respiratory chain dysfunction. MPV17 attenuated iron overload-induced myocardial ferroptosis through maintaining the mtGSH levels.

### 2.4. SLC25A10 Prevented Myocardial Ferroptosis by Mediating mtGSH Import

**Figure 4:**
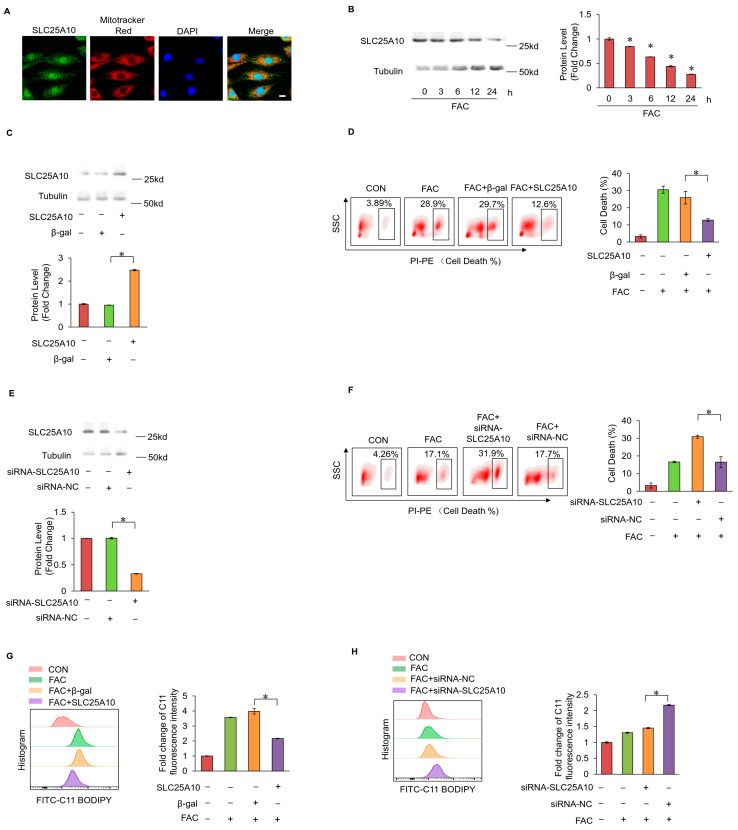
(**A**) The localization of SLC25A10 detected by immunofluorescence and confocal imaging; green, SLC25A10; red, mitotracker; blue, DAPI; scale bar, 20 µm; (**B**) The protein levels of SLC25A10 in cardiomyocytes treated with FAC for indicated time was evaluated by WB; (**C**) the overexpression of SLC25A10 by pcDNA3.1 eukaryotic vector in cardiomyocytes; (**D**) cell death labeled with PI staining was detected in each group; (**E**) the knockdown of SLC25A10 by siRNA in cardiomyocytes; (**F**) cell death labeled with PI staining was detected in each group; (**G**) fold changes in the fluorescence intensity of the C11-BO staining in mitochondria in each group; (**H**) fold changes in the fluorescence intensity of the C11-BO staining in mitochondria in each group; *, *p* < 0.05 was statistically significant.

SLC25A10 is a mitochondrial inner membrane GSH transporter that enables the import of GSH from the cytosol into the mitochondrial matrix. The immunofluorescence staining results demonstrated that SLC25A10 was localized in the mitochondria (Figure 4A). Additionally, the decreased expression levels of SLC25A10 were observed under the treatment of FAC, suggesting that SLC25A10 was involved in myocardial ferroptosis induced by iron overload (Figure 4B). The overexpression of SLC25A10 was performed in cardiomyocytes to examine its role in iron overload-induced myocardial ferroptosis (Figure 4C). The results showed that the enhanced expression of SLC25A10 prevented myocardial ferroptosis induced by FAC (Figure 4D). Conversely, silencing SLC25A10 through siRNA facilitated myocardial ferroptosis (Figure 4E,F). Subsequently, lipid peroxide accumulation was detected in cardiomyocytes. The overexpression of SLC25A10 decreased lipid peroxide accumulation in cardiomyocytes treated with FAC (Figure 4G). Conversely, the silencing of SLC25A10 led to increased lipid peroxide accumulation in cardiomyocytes treated with FAC (Figure 4H). This evidence demonstrated that SLC25A10 prevented iron overload-induced myocardial ferroptosis.

### 2.5. MPV17 Prevented Myocardial Ferroptosis through Maintaining the Protein Stability of SLC25A10

**Figure 5:**
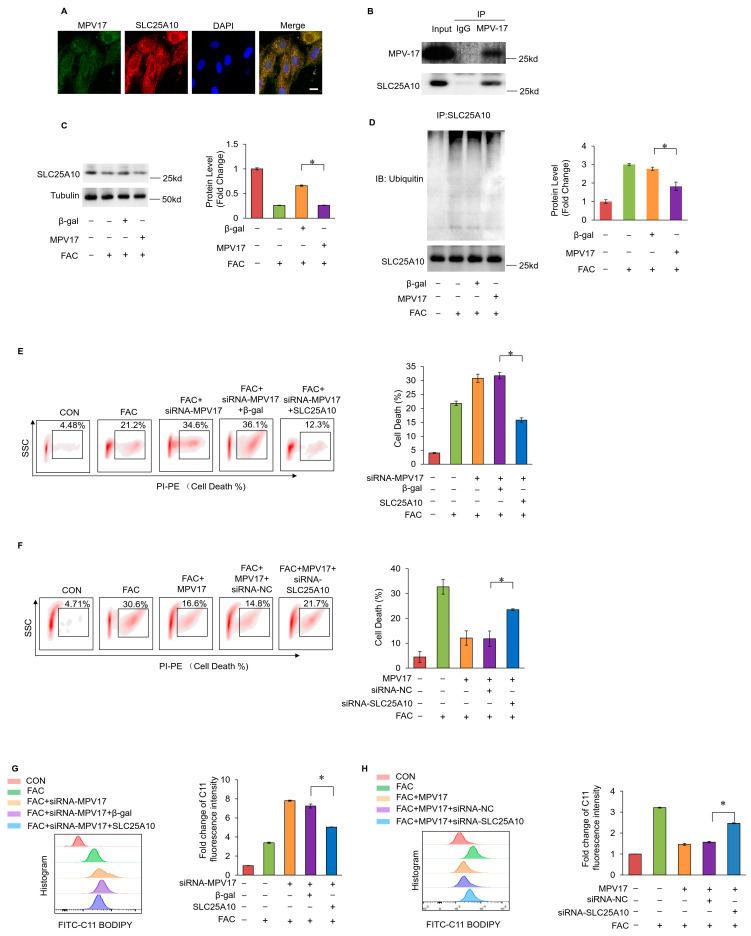
(**A**) The colocalization of MPV17 and SLC25A10 detected by immunofluorescence and confocal imaging; green, MPV17; red, SLC25A10; blue, DAPI; scale bar, 20 µm; (**B**) immunoprecipitation was performed to detect the interaction of endogenous MPV17 with SLC25A10 in cardiomyocytes; (**C**) the expression of SLC25A10 were evaluated by WB in each group; (**D**) WB was performed to detect the ubiquitination of SLC25A10; (**E**) the ratio of cell death labeled with PI staining in each group; (**F**) the ratio of cell death labeled with PI staining in each group; (**G**) fold changes in the fluorescence intensity of the C11-BO staining in each group; (**H**) fold changes in the fluorescence intensity of the C11-BO staining in each group; *, *p* < 0.05 was statistically significant.

We hypothesized that MPV17 and SLC25A10 had a functional connection in regulating myocardial ferroptosis. MPV17 was found to colocalize with SLC25A10 in cardiomyocytes (Figure 5A). The co-immunoprecipitation results showed that MPV17 could interact with SLC25A10 (Figure 5B). FAC treatment decreased the expression levels of SLC25A10, while the simultaneous overexpression of MPV17 partially recovered the expression levels of SLC25A10 (Figure 5C). Further studies revealed that FAC treatment led to protein instability and the ubiquitination of SLC25A10. The simultaneous overexpression of MPV17 reduced the ubiquitination of SLC25A10 (Figure 5D). These results indicated that MPV17 could stabilize SLC25A10 through interacting with it. Moreover, the knockdown of MPV17 aggravated myocardial ferroptosis induced by FAC, while the simultaneous overexpression of SLC25A10 attenuated myocardial ferroptosis (Figure 5E). The overexpression of MPV17 attenuated the myocardial ferroptosis induced by FAC, while the simultaneous knockdown of SLC25A10 aggravated these myocardial ferroptosis (Figure 5F). Next, we detected the lipid peroxide levels in FAC-treated cardiomyocytes. The knockdown of MPV17 increased lipid peroxide accumulation induced by FAC, while the simultaneous overexpression of SLC25A10 attenuated lipid peroxide accumulation (Figure 5G). The overexpression of MPV17 decreased lipid peroxide accumulation, while the simultaneous knockdown of SLC25A10 increased lipid peroxide accumulation (Figure 5H). These results indicated that SLC25A10 functioned downstream of MPV17 to regulate iron overload-induced myocardial ferroptosis. Taken together, MPV17 prevented iron overload-induced ferroptosis through maintaining the protein stability of SLC25A10.

### 2.6. Nrf2 Prevented Iron Overload-Induced Myocardial Ferroptosis through Transcriptionally Activating MPV17

**Figure 6:**
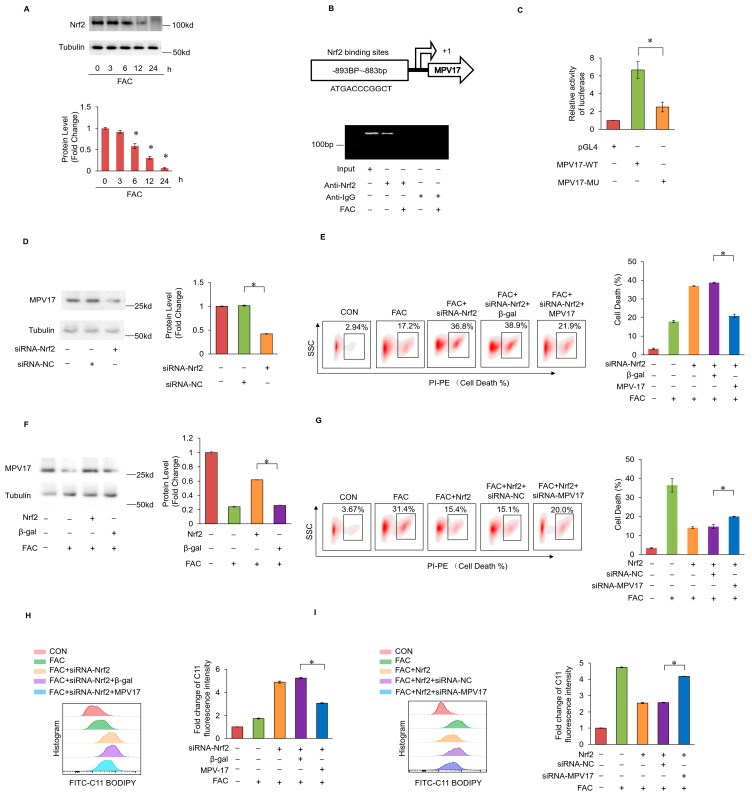
(**A**) The expression levels of Nrf2 in cardiomyocytes treated with FAC for the indicated time was evaluated by WB; (**B**) the 5′-flanking sequence of MPV17 contained a potential Nrf2 binding site; a Chip assay was performed to examined the binding site of Nrf2 in MPV17 promotor region; (**C**) luciferase activity was measured in HEK-293 cells transfected with pGL4-MPV17-WT or pGL4-MPV17-MU along with the Nrf2 overexpression constructs; (**D**) the expression of MPV17 in cardiomyocytes were evaluated by WB; (**E**) the percentage of cell death labeled with PI staining; (**F**) the expression of MPV17 in cardiomyocytes were evaluated by WB; (**G**) the percentage of cell death labeled with PI staining in each group; (**H**) fold changes in the fluorescence intensity of the C11-BO staining in each group; (**I**) fold changes in the fluorescence intensity of the C11-BO staining in each group; *, *p* < 0.05 was statistically significant.

Nrf2 is a core transcriptional factor in preventing ferroptosis through encoding antioxidant genes. Under the treatment of FAC, the expression levels of Nrf2 were decreased in cardiomyocytes (Figure 6A). An Nrf2 binding site was predicted to be in the promoter region of MPV17 by the JASPAR database, which was confirmed by the Chip assays and the luciferase assays (Figure 6B,C). Moreover, the knockdown of Nrf2 decreased the expression levels of MPV17 (Figure 6D), while the overexpression of Nrf2 partially recovered the expression levels of MPV17 in cardiomyocytes treated with FAC (Figure 6F). Next, we explored the functional relationship between Nrf2 and MPV17 in myocardial ferroptosis regulation. The knockdown of Nrf2 promoted myocardial ferroptosis, while the simultaneous overexpression of MPV17 attenuated the myocardial ferroptosis induced by FAC (Figure 6E). The overexpression of Nrf2 prevented myocardial ferroptosis, while the simultaneous knockdown of MPV17 promoted myocardial ferroptosis (Figure 6F). Then, we detected the lipid peroxide levels in FAC-treated cardiomyocytes. The knockdown of Nrf2 increased the lipid peroxide accumulation induced by FAC, while the simultaneous overexpression of MPV17 attenuated lipid peroxide accumulation (Figure 6G). The overexpression of Nrf2 decreased lipid peroxide accumulation, while the simultaneous knockdown of MPV17 increased lipid peroxide accumulation (Figure 6H). These results indicated that Nrf2 prevented iron overload-induced myocardial ferroptosis through transcriptionally activating MPV17.

## 3. Discussion

Ferroptosis is a newly identified programmed cell death process and plays an active role in several pathological processes [20,35,36,37]. Although myocardial ferroptosis has been implicated in ischemic cardiac diseases, the underlying mechanisms remain largely unknown. Clinical observations indicated that iron overload occurs in the cardiac ischemic zone, which is considered a dangerous factor in ferroptosis initiation [15]. This study further confirmed the role of iron overload in myocardial ferroptosis initiation during cardiac I/R injury. Moreover, we found an emerging role of MPV17 in protecting cardiomyocytes from iron overload-induced ferroptosis and attenuating cardiac ischemia/reperfusion (I/R) injury through maintaining the protein homeostasis of SLC25A10, which imports GSH from the cytosol into the mitochondria. Nrf2 can prevent myocardial ferroptosis through transcriptionally activating MPV17. This study revealed the Nrf2-MPV17-SLC25A10/mitochondrial glutathione (mtGSH) signaling pathway in myocardial ferroptosis regulation and provided new targets for the treatment of cardiac diseases. 

MPV17 is widely expressed in various tissues and organs, with its role little known. The close relationship between MPV17 mutation and human diseases has attracted broad research interests. Recent studies found that MPV17 actively participates in mitochondrial respiration and ROS metabolism as a scaffold protein [24,38,39]. In this study, MPV17 was indicated to participate in the regulation of mtGSH import. MPV17 has been demonstrated to be a transporter protein and form a nonselective channel in the mitochondrial inner membrane [24,40,41]. However, in this study, the regulation of mtGSH import by MPV17 did not depend on its channel function. MPV17 functioned more like a scaffolder protein to interact with the mtGSH transporter SLC25A10. This interaction protected SLC25A10 from ubiquitination and degradation. The absence of MPV17 induced by iron overload led to SLC25A10 instability and mtGSH exhaustion, leading to the final myocardial ferroptosis. These findings not only reveal the molecular mechanism of ferroptosis initiation but also enrich the functional role of MPV17.

Iron overload occurred during both the cardiac ischemic injury and the heart transplant process [8,37]. Mitochondria are an essential organelle in intracellular iron metabolism and play an indispensable role in ferroptosis initiation in several cases [42]. In human HT-1080 cells, the elimination of mitochondria protected cells from ferroptosis induction [25]. In DOX-induced myocardial ferroptosis, iron-chelator-targeting mitochondrial Fe^2+^ prevented myocardial ferroptosis initiation [43]. Free Fe^2+^ promoted lipid peroxide accumulation and ferroptosis initiation through a Fenton reaction [44,45]. The knockdown or overexpression of MPV17 had no effect on the levels of mitochondrial iron, although the overexpression of MPV17 diminished lipid peroxide accumulation in mitochondria. Lipid peroxide accumulation depended on not only the increased lipid oxidation but also the dysfunction of the lipid oxidation scavenging system. Our study showed that iron overload could delete the mtGSH and change the redox state of mitochondria, leading to lipid peroxide accumulation. However, the cause of iron overload during cardiac injury has not been further explored in this study.

In cysteine-deprivation-induced ferroptosis, mtGSH is essential for the maintenance of mitochondrial bioenergetic respiration and mitochondrial ROS metabolism [29]. GSH is also an important partner of GPX4, which converts peroxidized phosphatidylethanolamines (PEs) to hydroperoxyl-Pes [46]. GSH deletion would lead to the dysfunction of GPX4 and lipid peroxide accumulation. In this work, decreased mtGSH levels were observed, and the dysfunction of the GSH/GPX4 lipid oxidation scavenging system was responsible for myocardial ferroptosis initiation. Pre-treatment with GSH attenuated myocardial ferroptosis, while the simultaneous overexpression of mito-GPX4 further prevented myocardial ferroptosis. Moreover, we also found that glutathionylation insufficiency of the mitochondrial protein occurred because of mtGSH deletion. The glutathionylation of the mitochondrial protein usually protects them from oxidative damage under stress conditions [47]. The overexpression of MPV17 partially recovered the glutathionylation of the mitochondrial protein, including the key enzymes (nox4 and cox1) involved in the mitochondrial respiratory chain. These findings might explain the facts that the activities of mitochondrial respiration chain were decreased during iron overload-induced myocardial ferroptosis, while the overexpression of MPV17 could partially recover these activities. However, the relationship between mitochondrial protein glutathionylation and ferroptosis initiation has not been further explored in this study.

Nrf2 has long been considered an important transcriptional regulator in eliminating lipid peroxides, free iron and cystine import, and GSH production though its targeted genes [48,49]. However, the transcriptional activity of Nrf2 also depends on the cell types and physical conditions. This study identified MPV17 as a new target of Nrf2 in the regulation of iron overload-induced myocardial ferroptosis. These findings further revealed the mechanisms of Nrf2 in myocardial ferroptosis regulation and enriched the knowledge of molecular biology in cardiac diseases.

However, there were still limitations in this study. Firstly, the role of the mitochondrial respiratory chain was essential for ferroptosis initiation in several cases. Although we observed a decreased activity of the mitochondrial respiratory chain during iron overload-induced myocardial ferroptosis, the role of mitochondrial ROS from the respiratory chain in myocardial ferroptosis initiation was not further explored in this study. Secondly, MPV17 had multiple roles, maintaining proper mitochondrial functions under stress conditions. In our work, we mainly focused on its role in mtGSH regulation. Finally, the regulation mechanisms of mtGSH pool were a complex system, which was not fully examined in this study.

## 4. Materials and Methods

### 4.1. Cell Treatment

H9c2 cells (American Type Culture Collection) were cultured in the cell incubator with DMEM/F-12 (Gibco, 21331046, NewYork, NY, USA), containing 10% fetal bovine serum with 5% CO_2_ at 37 °C. Cells were co-cultured with 500 µM ammonium ferric citrate (FAC) to induce robust cell death. Cells were co-cultured with 100 µM FAC to induce mild cell death.

### 4.2. Mice Ischemia/Reperfusion Model

Mice underwent ligation with LAD for 30 min and were re-perfused for 3 H. Mice (*n* = 5/group) received a Propidium Iodide (PI) injection (10 mg/kg) (Solarbio, P8080, Beijing China) at the end of the surgery to label the ferroptotic cells. The cardiac tissues were frozen with liquid nitrogen and 5 μm sections were cut with an ice slicer (Leica, CM3050S, Heidelberg, Gemerny) and counterstained with DAPI to quantitatively detect cell death. The diastolic left ventricular internal diameters (LVIDd), the ejection fraction of the left ventricular diameter (EF) and the fractional shortening of the left ventricular diameter (FS) were measured by echocardiograph. All the approaches involving animals were approved by the Ethics Committee of Qingdao Agricultural University.

### 4.3. Detection of Myocardial Ferroptosis by PI Staining and Flowcytometry

The H9c2 cells were cultured with the treatment of FAC at the indicated times. The cells were digested with pancreatic enzyme (0.25%) for 2 min and resuscitated with PBS in a 1.5 mL tube. The supernatant was discarded and PI staining buffer (10 ug/mL) was added into the H9c2 cells. The cells were kept out of light and incubated in an ice bath for 30 min. After three washes with PBS, we used a BD FACS Aria III flow cytometer (BD, New York, NY, USA) and the PE channel to detect the PI positive cells.

### 4.4. Immunofluorescence Staining

The H9c2 cells cultured on the slide were fixed at 4 °C overnight and goat serum was used for blocking. The blocking buffer was discarded and the diluted antibody was added at the indicated dilution. We incubated the cells at 4 °C overnight. The secondary antibody labeled with FITC or Cy3 was added into the cells after three washes of PBST. The nuclear was labeled with DAPI. Images were captured with a Leica SP5 confocal microscope (Heidelberg, Gemerny). The anti-MPV17 antibody was from Proteintech (10310-1-AP, NewYork, USA), and the anti-SLC25A10 antibody was from Proteintech (12086-1-AP, NewYork, USA).

### 4.5. Prussian Blue Staining and Iron Level Detection

Prussian Blue staining was performed following the instructions (Solarbio, G1420, Beijing China). The free iron was detected following the instructions (Biolab, SNM159, Beijing China).

### 4.6. Masson Staining and Detection of Myocardial Fibrosis

Myocardial fibrosis was detected by a Masson Staining kit, following the instructions (Solarbio, G1340, Beijing China).

### 4.7. Detection of Mitochondrial Respiratory Chain Activity

Isolated mitochondria were used to measure the activity of Complex I-V, following the instruction of the detection kit (Solarbio BC0515, Solarbio BC3230, Solarbio BC3240, Solarbio BC0945, Solarbio, BC1440, Beijing China) by using the spectrophotometer/microplate reader(Biotech, Beijing, China).

### 4.8. Detection of Lipids Peroxides in the Cells

The C11 BODIPY 581/591 (C11-BO) staining dye was purchased from Invitrogen (Invitrogen, Thermo, Carlsbad, CA, USA). The cultured H9c2 cells were plated on a 6-well plate or on the slide. After treatment with the FAC, the C11-BO staining dye (2 µM) was added into the cultured cells, which were kept in 37 °C, 5% CO_2_ for 30 min. Then, the cells were digested with pancreatic enzyme (0.25%) and collected for detection. A BD FACS Aria III flow cytometer was used to detect the fluorescence signaling. FITC channel and PE channel were used for the detection of green fluorescence and red fluorescence, respectively. The cultured cells plated on the slide were detected by a Leica SP5 confocal microscope directly after the staining.

### 4.9. Western Blotting (WB) Assay

The procedures for WB assays were described in the previous work. The anti-α-actinin antibody was from Sigma (A7732, Rahway, NJ, USA); the anti-β-Tubulin antibody was from Abclonal (A17073, Wuhan, China); the anti-ACSL4 antibody was from BOSTER (A04372-2); the anti-GPX4 antibody was from BOSTER (A02059-1); the anti-Fth1 antibody was from BOSTER (BM4487); the anti-NOX4 antibody was from BOSTER (BM4135, Wuhan, China); the anti-COX-1 antibody was from BOSTER (PB9002); the anti-Nrf2 antibody was from Proteintech (16396-1-AP, New York, NY, USA); and the anti-GSH antibody was from VIROGEN (101A, New York, NY, USA). The anti-Ubiquitin antibody was from Santacruz (sc-8017, Santa Cruz, CA, USA). The β-tubulin antibody was used for loading control.

### 4.10. Immunoprecipitation Assay

The immunoprecipitation assay was performed as previously described [50]. The RIPA buffer with a cocktail (Merk, 539134, Rahway, NJ, USA) was used for protein lysis. The 2µg antibody and 50 µL protein A/G Agarose beads (Biolinkedin, Ik-1004, Wuhan, China) were used for one sample. The mixtures were slightly rotated at 4 °C overnight. The immunoprecipitants were resuspended in sample buffer with sodium lauryl sulfate and boiled at 98 °C for 10 min.

### 4.11. Cell Transfection

The siRNA and plasmid vectors were transfected with lippo3000 following the manufacturer’s instructions. The siRNA targeting MPV17 were purchased from Origene (SR508989, Seattle, WA, USA); the siRNA targeting slc25a10 were purchased from Origene (SR509035); the siRNA sequences targeting Nrf2 were 5′-UUGUGUUCAGUGAAAUGCCGG-3′, which was validated previously; the open reading frame (ORF) of SLC25A10 (rat) or Nrf2 (rat) was constructed into the eukaryotic vectors pcDNA3.1.

### 4.12. Detection of Mitochondrial Outer Membrane Potential

JC-1 was added into the H9c2 cells and incubated for 30 min in 37 °C. After three washes, a BD FACS Aria III flow cytometer was used to detect the red-green fluorescence ratio through the FITC channel and the PE channel, respectively.

### 4.13. Detection of the mtGSH Levels

The mitochondria were isolated by the mitochondria isolation kit following the instructions (Solarbio, SM0020, Beijing, China). Then, the mtGSH levels were detected by GSH detection kit following the instructions (Nanjing Jiancheng, A006-2-1, Nanjing, China).

### 4.14. Detection of the Luciferase Activity

The instructions for the luciferase assay were described previously [51].

### 4.15. Chip Assay

The well-cultured cardiomyocytes were immersed in 1% formaldehyde solution and treated under vacuum for 10 min to promote cross-linking. A total of 125 mm glycine was applied to interrupt the cross-linking reaction. After three washes with cooled PBS, the lysis buffer containing protease inhibitor was used for cell lysis. The collected samples were processed by ultrasonic crusher to lyse the cells and release chromatin, which was sheared into 100–1000 BP fragments using an ultrasonic crusher. The un-lysed cell fragments were removed and antibody incubation was performed at 4 °C overnight. The magnetic beads were applied for the binding of the antibody at room temperature for 1 h. Low-salt, high-salt, lithium-chloride and TE washing buffer were used to wash the beads sequentially to remove non-specific binding. A total of 200 mM NaCl was used for reverse cross-linking at 65 °C for 4 h. After digestion with proteinase K at 45 °C for 1 h, the mixtures were purified by using phenol chloroform. A (BIO-RAD) T100 Thermal Cycler (Biorad, Berkeley, CA, USA) was used for the replication of the aimed DNA fragments.

Chip-PCR primers: F: 5′-GCTAGCCGGGTCATTTTACA-3′, R:5′-CGGAGCTCAGACTTGTTCCA-3′.

### 4.16. Statistical Analysis

At least three independent experiments were performed. The GraphPad Prism 9.0 software was used for the data analysis. Shapiro–Wilk normality was performed. Student’s t-test was applied for the comparison of two groups. One-way ANOVA was performed for the comparison of three or more groups. Data are shown as the mean ± SD, *; *p* < 0.05 was statistically significant.

## 5. Conclusions

In conclusion, we demonstrated the important role of MPV17 in preventing iron overload-induced ferroptosis and delineated the Nrf2-MPV17-SLC25A10/mtGSH signaling pathway in the regulation of ferroptosis. These findings provide a new strategy for the treatment of cardiac diseases.

## Data Availability

No new data were created or analyzed in this study. Data sharing is not applicable to this article.

## Supplementary Materials

The following supporting information can be downloaded at: https://www.mdpi.com/article/10.3390/ijms251910832/s1.
