# Supplementary material for: MPV17 Prevents Myocardial Ferroptosis and Ischemic Cardiac Injury through Maintaining SLC25A10-Mediated Mitochondrial Glutathione Import
Source: Int J Mol Sci. 2024 Oct 9;25(19):10832. doi: 10.3390/ijms251910832 (PMC11476822; doi:10.3390/ijms251910832)
Supplement: Supplementary file 1 [file ijms-25-10832-s001.zip › ijms-3247564-supplementary.pdf]

International Journal of
Molecular Sciences

MDPI

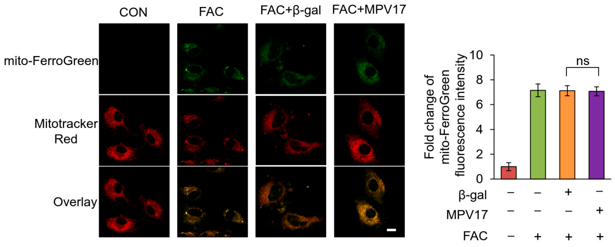

Supplementary Figure S1. Detection of mitochondrial Fe^{2+} levels with mito-FerroGreen; green, mito-FerroGreen; red, Mitotracker; ns, none significance.
